# Professional satisfaction of health professional undergraduates and influencing factors in Hebei province, China

**DOI:** 10.1186/s12909-021-02718-4

**Published:** 2021-05-14

**Authors:** Yatian Liu, Xiaofeng Sun, Yuqi Yuan, Ye Zhang, Jieru Liu, Yufan Duan, Longmei Tang

**Affiliations:** 1 School of Public Health, Hebei Medical University, Shijiazhuang, Hebei China; 2 Hebei Province Key Laboratory of Environment and Human Health, Hebei Shijiazhuang, China

**Keywords:** Health professional students, Professional satisfaction, University

## Abstract

**Background:**

Professional satisfaction of health professional students can impact on their medical professional achievement. Understanding the professional satisfaction of health professional students and identifying its relative factors is beneficial to strengthen the professionalism education of health professional students, and provide solid foundation for future medical achievements.

**Methods:**

A self-made questionnaire was used to survey undergraduate students of six medical universities in Hebei province. The survey included three aspects: students’ basic situation, professional selection and cognition, and basic situation of colleges. The Kruskal–Wallis *H* test was used to compare the professional satisfaction of students with different characteristics. All covariates were used in the ordinal logistics regression analysis to identify the independent factors associated with professional satisfaction.

**Results:**

A total of 1238 (97.7%) students responded to the questionnaire in the survey, and 66.0% were women. Students with public health majors had decreased satisfaction compared with those with clinical-related majors. Professional satisfaction decreased among women compared with men. The non-first-choice students had lower professional satisfaction compared with the first-choice students. Students who chose their volunteer with the help of others had lower professional satisfaction compared with students who independently chose their volunteer. Students who did not understand the employment status had lower professional satisfaction compared with students who understood the employment status. Students with fewer employment prospects had lower professional satisfaction compared with students with bright employment prospects. Students generally dissatisfied with the canteen had lower professional satisfaction compared with students satisfied with the canteen. Students who were very satisfied or satisfied with teaching levels were more likely to have professional satisfaction.

**Conclusions:**

The professional satisfaction of health professional undergraduates in Hebei province is high. Employment-related aspects and university environment influence professional satisfaction including canteens, understanding of employment status, teachers’ teaching level, etc., which are the main factors affecting professional satisfaction, but the factors such as student employment prospects and majors cannot be changed in the current environment.

## Background

Professional satisfaction refers to how students’ experiences meet their expectations [1–3]. Studies showed the impact of professional satisfaction on their professional achievement. Professional satisfaction affects professional achievement by affecting employment confidence. When students’ professional satisfaction is high, they tend to be more confident in their future jobs [4, 5], have a more correct employment attitude [6, 7], and achieve better career prospects. Professional satisfaction also affects the level of career decision-making and career preparation behavior. The higher the level of career decision-making, the higher the level of preparation behavior related to a career path. The actual implementation of professional preparation behaviors in college life is essential to successfully enter the professional world after graduating from college [8]. Understanding the professional satisfaction of students helps effectively grasp the mental dynamics of modern students and make the education more effective [9]. It also helps identify the existing problems in the current education, improve the quality of education [10], and develop better health professional graduates.

China is the largest developing country in the world. Chinese medical technology and medical development level play a vital role in the world’s medical technology and medical development. Training outstanding health professional graduates is the key to improving medical standards. Compared with ordinary college students, health professional undergraduates study longer, have more onerous learning tasks, and experience more stress and depression [11]. Improving professional satisfaction is conducive to stimulating students’ positive attitudes and reducing mental illness induced by bad emotions [12, 13]. Improving professional satisfaction also enhances students’ sacred mission and sense of responsibility for the health profession, stimulates their own learning motivation, improves learning efficiency [14], and provides a solid foundation for future medical achievements.

Many factors affect professional satisfaction, including three major aspects: individual characteristics, social factors, and college factors. Individual characteristics comprise the basic personal information, including major, grade, sex, academic performance, and so forth. Previous studies found that different professional categories had a significant impact on the overall satisfaction of students. The lack of professional characteristics in professional positioning and curriculum setting leads to a poor sense of belonging and identity of students [15], thereby affecting the professional satisfaction. Among health professional students, the professional satisfaction of students of different health professional majors is not the same, and the salary and job safety in the public health field are lower [16, 17]. Therefore, student satisfaction is lower than that in other majors. In terms of sex, different studies reached different conclusions. Some studies showed that sex had statistically significant differences in the evaluation process of professional satisfaction. Male professional satisfaction was significantly higher than that of female students [18, 19]. Some studies showed that the scores of boys in all dimensions of school satisfaction were lower than those of girls [20]. Tang et al. showed that girls were less satisfied with science majors, while boys were less satisfied with language and other liberal arts majors [19]. In terms of grades, Tan et al. believe that students’ professional satisfaction had a significant relationship with the time they entered the school. The degree of professional satisfaction declined with the increase in the time when they entered the school, [21]. In terms of performance, study investment, employment expectations, and professional satisfaction were extremely significantly positively correlated [22].

Second are social factors, including students’ enrollment willingness, voluntary selection methods, career guidance courses, and professional prospects. The comprehensive strength, development prospects, and social recognition of colleges and universities have a greater impact on candidates applying for the school; professional employment and development prospects have a large impact on candidates’ choice of majors [23]. Noorafshan et al. showed that when candidates chose colleges and majors, the suggestions of parents and teachers had a great influence on candidates’ choices [24]. At the same time, the difference in professional satisfaction of different college entrance examination decision-makers was statistically significant; students whose majors were determined by themselves had higher satisfaction [10]. Different admission results after applying for the examination also affected students’ professional satisfaction. Students who were admitted by their first volunteer were more satisfied with their professions, while those admitted using the second volunteer, the third volunteer, obedience to distribution, and other methods were gradually less satisfied with their profession [10, 19]. Related studies showed that professional prospects also had a significant impact on student satisfaction. As confidence in employment prospects continued to increase, professional satisfaction also increased. Therefore, a significant positive correlation was found between employment prospects and professional satisfaction [25].

Third are the external environmental factors closely related to student life, such as the school environment (campus architecture, school appearance, school appearance, living facilities, etc.), school teachers, and teaching capabilities. Some studies pointed out that the degree of satisfaction with curriculum had the greatest influence on professional satisfaction [22]. Xu et al. found that the school environment had the most intuitive impact on student satisfaction. Among the top five index factors that caused a high level of satisfaction among vocational college students, three were related to the school environment [26]. Li et al. showed that the better the books and materials provided by the school, the stronger the learning atmosphere in the class, and the higher the school’s emphasis on majors, the higher the students’ satisfaction with the majors [27]. The teaching ability of teachers had a significant influence on the professional satisfaction of students. The knowledge reserve, expression ability, and logical thinking ability of teachers impacted satisfaction [28].

Most previous studies conducted surveys on the satisfaction of students in a certain school or a certain major, with a small survey scope and a small sample size [1, 12, 29–32]. Moreover, most of the factors that influenced students’ professional satisfaction were discussed from a relatively single aspect, while students’ personality characteristics and education and teaching were the main factors to be discussed [1, 2, 15, 29, 31–34]; also, the research on students’ life and environment was less involved. Therefore, a survey on health professional undergraduate professional satisfaction was conducted in all five medical schools enrolling undergraduate majors in Hebei province, and the relevant factors for professional satisfaction from personal characteristics, school major selection and cognition, and university environment were analyzed. The findings might help develop education in medical schools of China and provide strong evidence for other scholars to study the similarities and differences in health professional student education between China and other countries.

## Methods

### Setting

The survey was conducted among undergraduate students of six medical universities in Hebei province: Hebei Medical University, North China University of Science and Technology, Health Science Center of Hebei University, Hebei University of Chinese Medicine, Chengde Medical University, and Hebei North University.

### Sample size estimation

The sample size was estimated according to the sample size formula for estimating finite population proportion [35], and the minimum sample size was determined to be 1000. The profession satisfaction proportionπis 60%, with a significance levelαset at 0.05, and an allowable errorδof 3%. Taking into account the response rate and efficiency of the questionnaire, the final sample size was determined to be 1100.

### Study design

**Fig. 1:**
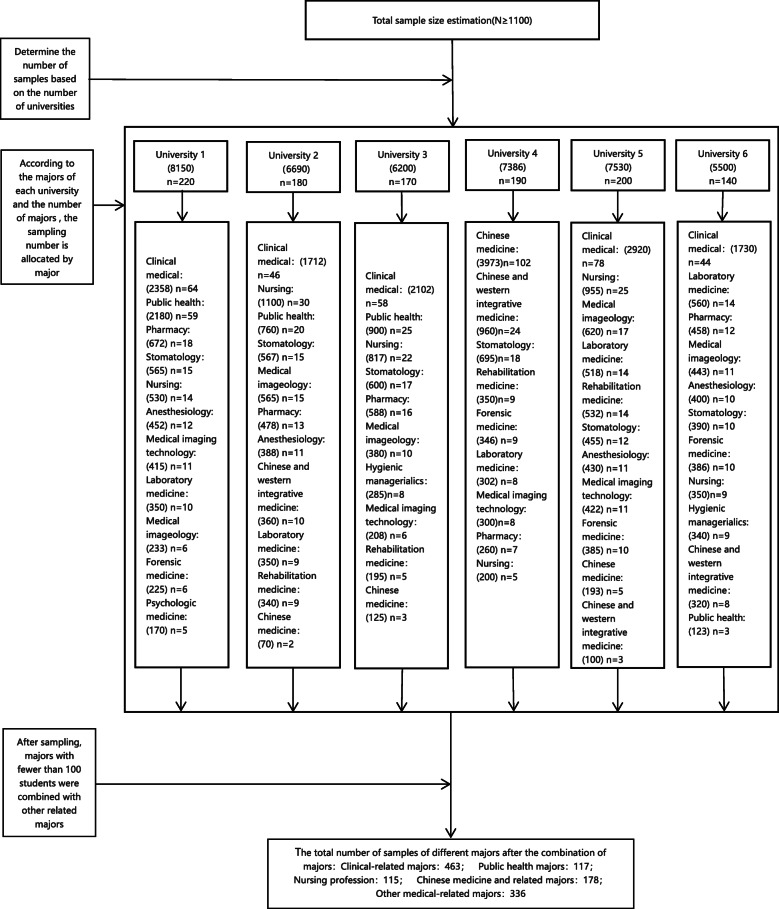
Sampling process flow chart. *The number in brackets is the total number of students in each university or major. **University 1 indicates Hebei Medical University; University 2 indicates North China University of Science and Technology; University 3 indicates Health Science Center of Hebei University; University 4 indicates Hebei University of Chinese Medicine; University 5 indicates Chengde Medical University; University 6 indicates Hebei North University. ***Clinical-related majors includes Clinical medical; Stomatology; Anesthesiology; Psychologic medicine; Chinese medicine and related majors includes Chinese and western integrative medicine; Chinese medicine; Other medical-related majors includes Pharmacy; Medical imaging technology; Laboratory medicine; Medical imageology; Forensic medicine; Rehabilitation medicine; Hygienic managerialics

A cross-sectional survey was performed between September 2018 and June 2019. A hierarchical cluster randomized sampling method was used to understand the majors of all undergraduate health professional colleges in Hebei province and the number of each major, and also proportionally allocate the number of surveys for each major. The survey also determined the number of administrative classes to be selected based on the assigned number of surveyed persons and the average number of administrative classes in the major. All undergraduate students in the selected administrative classes were invited to participate in this survey. After sampling, majors with fewer than 100 students were combined with other related majors. As shown in Fig. 1.

### Participants

All students in the selected classes were invited to participate in the survey, except those unwilling to participate. Students who were suspended from school, went out for internships, did not live in school, or underwent professional adjustments during the survey period were also excluded from the survey.

### Questionnaire

A structured questionnaire was used in the survey. The questionnaire comprised three sections:(1) characteristics of students, such as major, grade, sex, and grade ranking; (2) professional choice and cognition, such as professional selection method, whether had training experience of employment guidance course, employment prospects and types of study major; (3) degree of satisfaction with universities, such as university environment and living facilities (campus and surrounding environment, canteen, dormitory, sports and fitness, etc.), learning facilities (theory and experiment teaching equipment, library, self-study room), and teaching (textbooks, curriculum arrangement, teaching level, etc.).

The questionnaire used in the survey was developed on the basis of a literature review. Based on scientific and logical considerations, the entire questionnaire was discussed with team members including the students, teachers, clinicians, and managers.

The Cronbach’s alpha coefficient of the questionnaire was 0.884. The KMO value was 0.949, and Bartlett’s test of sphericity was passed with a significance level of 0.05. In the factor analysis, six principal components were extracted with the cumulative proportion of variance explained nearly 70%. It indicated that the questionnaire had good reliability and validity.

### Variables

The principal outcome of interest was professional satisfaction. The degree of satisfaction from low to high was very dissatisfied, dissatisfied, average, satisfied, and very satisfied. In this study, the number of students who were dissatisfied and were very dissatisfied was too small (38 and 12, respectively). The latter three categories (average, dissatisfied, and very dissatisfied) were merged to avoid the impact of uneven data distribution on the results. Finally, three categories were used in the analysis.

### Statistical analysis

The data were analyzed using the Statistical Package for Social Scientists (IBM SPSS version 25.0). The counts and proportions were used to describe categorical variables, and the Kruskal–Wallis *H* test was used to study the differences in satisfaction. All covariates were used in the ordinal logistics regression analysis. The results were presented as an adjusted odds ratio (ORadj) with a 95% confidence interval (CI). A two-sided *P* value < 0.05 was considered statistically significant.

### Ethics approval and consent to participate

This study was approved by the Medical Ethics Committee of Hebei Medical University (reference number: 2020205). All methods were performed in accordance with “ Ethical Review Measures for Biomedical Research Involving Human Subjects “ [36] and “Regulations of the Ethics Committee of Hebei Medical University”. Before filling out the questionnaire, the interviewees confirmed that they fully understood the precautions. Participation in the research was voluntary, and all participants provided informed consent.

## Results

Altogether 1238 students received the questionnaire, and 1209 (97.7%) completed it. Among participants, 34.0% were male; 54.6% were of second grade, 30.6% were of third grade, and 14.8% were of fourth or fifth grade; 38.3% were clinical students, 9.7% were public health students, 9.5% were nursing students, 14.7% were traditional Chinese medicine students, and 27.8% were other medical-related majors students.

Of all health professional students surveyed, 28.0% were very satisfied with their majors, and 44.1% were satisfied with their majors. The students whose major was public health, whose gender was female, and whose grade ranking was 51–75% had low satisfaction (Table 1).

**Table 1:** Professional satisfaction of students with different characteristics

Basic situation	Category	*n*	Mean rank	Number of very satisfied (%)	Number of satisfied (%)	Number of average and dissatisfied (%)	*Kruskal–Wallis H*	*P*
Major							28.404	< 0.001
	Clinical-related majors	463	554.95	148(32.0)	221(47.7)	94(20.3)		
	Public health majors	117	713.61	25(21.4)	38(32.5)	54(46.2)		
	Nursing profession	115	620.88	29(25.2)	53(46.1)	33(28.7)		
	Chinese medicine and related majors	178	588.61	51(28.7)	83(46.6)	44(24.7)		
	Other medical-related majors	336	639.40	86(25.6)	138(41.1)	112(33.3)		
Grade							3.886	0.143
	Second grade	660	592.52	195(29.5)	290(43.9)	175(26.5)		
	Third grade	370	632.81	86(23.2)	175(47.3)	109(29.5)		
	Fourth and fifth grade	179	593.53	58(32.4)	68(38.0)	53(29.6)		
Sex							18.047	< 0.001
	Male	411	549.54	151(36.7)	162(39.4)	98(23.8)		
	Female	798	633.57	188(23.6)	371(46.5)	239(29.9)		
Grade ranking*							10.042	0.018
	Top 25%	408	575.81	136(33.3)	164(40.2)	108(26.5)		
	26–50%	439	608.70	119(27.1)	198(45.1)	122(27.8)		
	51–75%	251	656.06	50(19.9)	122(48.6)	79(31.5)		
	Bottom 25%	111	582.18	34(30.6)	49(44.1)	28(25.2)		

*Grade ranking was divided into four groups basing on the rank of score mean in the administrative class during the previous academic year

The data in Table 2 shows that students who were not the first choice admitted, were chose by others to volunteer, did not participate in career guidance courses, had less professional prospects, and did not understand the employment status had low professional satisfaction.

**Table 2:** Professional satisfaction of health professional students with different professional selection methods and professional cognition

	Category	*n*	Mean rank	Number of very satisfied (%)	Number of satisfied (%)	Number of average and dissatisfied (%)	*Kruskal–Wallis H*	*P*
Admission volunteer							62.165	< 0.001
	First choice	672	538.96	244(36.3)	287(42.7)	141(21.0)		
	Non-first choice	537	687.64	95(17.7)	246(45.8)	196(36.5)		
Way of voluntary choice							82.312	< 0.001
	Oneself	619	522.01	225(36.3)	288(46.5)	106(17.1)		
	Others	590	692.07	114(19.3)	245(41.5)	231(39.2)		
Career guidance courses							19.215	< 0.001
	Participate	1080	590.81	315(29.2)	487(45.1)	278(25.7)		
	Did not participate	129	723.84	24(18.6)	46(35.7)	59(45.7)		
Professional prospects							145.458	< 0.001
	Bright	672	503.99	259(38.5)	311(46.3)	102(15.2)		
	Bleak	537	731.41	80(14.9)	222(41.3)	235(43.8)		
Employment status							82.651	< 0.001
	Understand	249	437.75	135(54.2)	75(30.1)	39(15.7)		
	Did not understand	960	648.38	204(21.3)	458(47.7)	298(31.0)		

Table 3 shows that those who were satisfied with the campus environment and surrounding environment, dormitories, canteens, physical fitness facilities, theoretical teaching equipment, experimental teaching equipment, libraries, self-study rooms, teaching materials, curriculum arrangements, and teachers’ teaching level had higher professional satisfaction.

**Table 3:** College situation and students’ professional satisfaction

College situation	Category	*n*	Mean rank	Number of very satisfied (%)	Number of satisfied (%)	Number of average and dissatisfied (%)	*KruskalWallis H*	*P*
Campus environment							220.369	< 0.001
	Very satisfied	278	369.09	175(62.9)	79(28.4)	24(8.6)		
	Satisfied	363	600.99	77(21.2)	213(58.7)	73(20.1)		
	Average and dissatisfied	568	723.02	87(15.3)	241(42.4)	240(42.3)		
Around the school							167.611	< 0.001
	Very satisfied	268	383.01	167(62.3)	71(26.5)	30(11.2)		
	Satisfied	289	623.87	56(19.4)	165(57.1)	68(23.5)		
	Average and dissatisfied	652	687.88	116(17.8)	297(45.6)	239(36.7)		
Dormitory							162.503	< 0.001
	Very satisfied	252	382.71	155(61.5)	71(28.2)	26(10.3)		
	Satisfied	308	605.77	72(23.4)	164(53.2)	72(23.4)		
	Average and dissatisfied	649	690.95	112(17.3)	298(45.9)	239(36.8)		
Canteen							210.595	< 0.001
	Very satisfied	226	341.63	153(67.7)	57(25.2)	16(7.1)		
	Satisfied	311	583.39	71(22.8)	185(59.5)	55(17.7)		
	Average and dissatisfied	672	703.57	115(17.1)	291(43.3)	266(39.6)		
Textbook							212.199	< 0.001
	Very satisfied	340	399.45	192(56.5)	117(34.4)	31(9.1)		
	Satisfied	453	633.66	87(19.2)	250(55.2)	116(25.6)		
	Average and dissatisfied	416	741,79	60(14.4)	166(39.9)	190(45.7)		
Teachers’ teaching level							237.239	< 0.001
	Very satisfied	394	404.51	217(55.1)	142(36.0)	35(8.9)		
	Satisfied	467	662.67	72(15.4)	262(56.1)	133(28.5)		
	Average and dissatisfied	348	754.61	50(14.4)	129(37.1)	169(48.6)		
Theory course arrangement							239.662	< 0.001
	Very satisfied	372	399.63	208(55.9)	132(35.5)	32(8.6)		
	Satisfied	453	643.27	83(18.3)	248(54.7)	122(26.9)		
	Average and dissatisfied	384	758.80	48(12.5)	153(39.8)	183(47.7)		
Experimental course arrangement							221.438	< 0.001
	Very satisfied	362	404.76	203(56.1)	123(34.0)	36(9.9)		
	Satisfied	425	633.45	80(18.8)	238(56.0)	107(25.2)		
	Average and dissatisfied	422	748.12	56(13.3)	172(40.8)	194(46.0)		
Theory teaching equipment							231.137	< 0.001
	Very satisfied	318	381.06	191(60.1)	100(31.4)	27(8.5)		
	Satisfied	425	625.26	78(18.4)	250(58.8)	97(22.8)		
	Average and dissatisfied	466	739.34	70(15.0)	183(39.3)	213(45.7)		
Experimental teaching equipment							230.275	< 0.001
	Very satisfied	313	376.08	189(60.4)	100(31.9)	24(7.7)		
	Satisfied	430	632.13	74(17.2)	256(59.5)	100(23.3)		
	Average and dissatisfied	466	733.72	76(16.3)	177(38.0)	213(45.7)		
Physical fitness facilities							235.271	< 0.001
	Very satisfied	228	336.30	157(68.9)	55(24.1)	16(7.0)		
	Satisfied	303	564.10	80(26.4)	172(56.8)	51(16.8)		
	Average and dissatisfied	678	713.64	102(15.0)	306(45.1)	270(39.8)		
Self-study room							232.706	< 0.001
	Very satisfied	292	367.02	183(62.7)	86(29.5)	23(7.9)		
	Satisfied	337	607.08	72(21.4)	192(57.0)	73(21.7)		
	Average and dissatisfied	580	723.61	84(14.5)	255(44.0)	241(41.6)		
Library							180.348	< 0.001
	Very satisfied	360	430.26	191(53.1)	123(34.2)	46(12.8)		
	Satisfied	361	604.57	81(22.4)	200(55.4)	80(22.2)		
	Average and dissatisfied	488	734.22	67(13.7)	210(43.0)	211(43.2)		

As shown in Table 4, satisfaction of students with public health majors was more likely to decrease compared with the satisfaction of those clinical-related majors [ORadj (95% CI): 1.81 (1.15–2.85)]. Professional satisfaction was more likely to decrease in women compared with men [ORadj (95% CI): 1.33 (1.03–1.72)]. Non-first-choice students were more likely to have lower professional satisfaction compared with the first-choice students [ORadj(95% CI): 1.37 (1.07–1.75)]. Students who chose their volunteer with the help of others were more likely to have low professional satisfaction compared with students who independently chose their volunteer [ORadj (95% CI): 1.79 (1.40–2.28)]. Students who did not understand the employment status were more likely to have lower professional satisfaction compared with students who understood the employment status [ORadj (95% CI): 2.14 (1.56–2.93)]. Students with bleak employment prospects were more likely to have lower professional satisfaction compared with students with bright employment prospects [ORadj (95% CI): 2.40 (1.86–3.08)]. Students who were satisfied with the canteen were more likely to have low professional satisfaction compared with students who were very satisfied with the canteen [ORadj (95% CI): 1.80 (1.04–3.12)]. Students who were generally dissatisfied with the canteen were more likely to have lower professional satisfaction compared with students who were satisfied with the canteen [ORadj (95% CI): 2.45 (1.38–4.35)]. Students who were very satisfied or satisfied with teaching levels were more likely to have professional satisfaction [ORadj (95% CI): 2.15 (1.25–3.70); ORadj (95% CI): 1.64 (0.85–3.19)].

**Table 4:** Association factors of professional satisfaction: logistic regression

	β	*χ*2	*P*	OR	95% Cl	
					Lower	Upper
Major						
Clinical-related majors						
Public health majors	0.595	6.628	0.010	1.81	1.15	2.85
Nursing profession	0.220	0.988	0.320	1.25	0.81	1.92
Chinese medicine and related majors	0.048	0.067	0.796	1.05	0.73	1.51
Other medical-related majors	0.252	2.619	0.106	1.29	0.95	1.74
Sex						
Male						
Female	0.287	4.842	0.028	1.33	1.03	1.72
Grades ranking						
Top 25%						
26–50%	−0.004	0.001	0.978	1.00	0.75	1.32
51–75%	0.280	2.772	0.096	1.32	0.95	1.84
Bottom 25%	−0.114	0.260	0.610	0.89	0.58	1.38
Grade						
Second grade						
Third grade	0.005	0.001	0.970	1.01	0.77	1.32
Fourth and fifth grade	0.168	0.865	0.352	1.18	0.83	1.69
Admission volunteer						
First choice						
Non-first choice	0.312	6.030	0.014	1.37	1.07	1.75
Way to choose major						
Oneself						
Others	0.580	21.353	< 0.001	1.79	1.40	2.28
Career guidance courses						
Participate						
Did not participate	0.192	0.960	0.327	1.21	0.83	1.78
Employment status						
Understand						
Did not understand	0.760	22.208	< 0.001	2.14	1.56	2.93
Employment prospects						
Bright						
Bleak	0.874	46.121	< 0.001	2.40	1.86	3.08
Campus environment						
Very satisfied						
Satisfied	0.258	1.032	0.310	1.30	0.79	2.13
Average and dissatisfied	0.424	2.408	0.121	1.53	0.90	2.61
Around the school						
Very satisfied						
Satisfied	0.291	1.298	0.255	1.34	0.81	2.21
Average and dissatisfied	0.204	0.694	0.405	1.23	0.76	1.98
Dormitory						
Very satisfied						
Satisfied	−0.161	0.341	0.559	0.85	0.50	1.46
Average and dissatisfied	−0.476	2.758	0.097	0.62	0.35	1.09
Canteen						
Very satisfied						
Satisfied	0.586	4.355	0.037	1.80	1.04	3.12
Average and dissatisfied	0.897	9.367	0.002	2.45	1.38	4.35
Textbook						
Very satisfied						
Satisfied	−0.186	0.502	0.478	0.83	0.50	1.39
Average and dissatisfied	−0.141	0.209	0.648	0.87	0.48	1.59
Teachers’ teaching level						
Very satisfied						
Satisfied	0.765	7.635	0.006	2.15	1.25	3.70
Average and dissatisfied	0.497	2.160	0.142	1.64	0.85	3.19
Theory course arrangement						
Very satisfied						
Satisfied	0.154	0.208	0.648	1.17	0.60	2.24
Average and dissatisfied	0.451	1.279	0.258	1.57	0.72	3.43
Experimental course arrangement						
Very satisfied						
Satisfied	−0.107	0.150	0.698	0.90	0.52	1.54
Average and dissatisfied	−0.097	0.089	0.765	0.91	0.48	1.71
Theory teaching equipment						
Very satisfied						
Satisfied	0.525	2.491	0.114	1.69	0.88	3.25
Average and dissatisfied	0.498	1.835	0.176	1.65	0.80	3.38
Experimental teaching equipment						
Very satisfied						
Satisfied	0.063	0.036	0.849	1.07	0.55	2.05
Average and dissatisfied	0.060	0.025	0.876	1.06	0.50	2.24
Physical fitness facilities						
Very satisfied						
Satisfied	−0.218	0.497	0.481	0.80	0.44	1.48
Average and dissatisfied	0.156	0.248	0.618	1.17	0.63	2.16
Self-study room						
Very satisfied						
Satisfied	0.378	1.867	0.172	1.46	0.85	2.51
Average and dissatisfied	0.553	3.827	0.050	1.74	1.00	3.02
Library						
Very satisfied						
Satisfied	−0.291	1.767	0.184	0.75	0.49	1.15
Average and dissatisfied	0.088	0.138	0.711	1.09	0.69	1.74

## Discussion

### Findings and interpretation

As one of the major foundations of the university, university students constitute the main structure of different organizations and systems in the community. The satisfaction of all activities in the university can affect their viewpoints about their educational field and then influence the study motivation and study quality [32].

In this survey, 28.0% of the health professional undergraduates in Hebei province were very satisfied with their professional satisfaction, and 44.1% were satisfied. These two situations were combined as “achieved satisfaction,” that is to say, 72.1% of the students achieved satisfaction with their studies, which was higher than other similar research results [25, 37–41]. One reason was that medical professionals had a high social status [42, 43], as the medical profession was a high-income field internationally [44]. High social status and income made students opt for medical studies and hence be more satisfied with their profession. The other reason was a large demand for medical professionals in the future. Students with medical background had better employment prospects [45]. It made health professional undergraduate students achieve higher professional satisfaction compared with other non-health professional undergraduate students. In the survey, the professional satisfaction of health professional undergraduate students in Hebei province was also higher than that of students in other provinces [41]. It might be related to the employment guidance courses conducted in all universities in Hebei province. The employment guidance courses promoted students to understand their major and employment prospects, deepened professional beliefs, and finally increased their professional satisfaction.

Many factors influenced students’ professional satisfaction. The first was canteen services, it has the biggest influence on professional satisfaction. Living facilities in universities were an indispensable part of university construction [46]. The daily activities of students were concentrated on campus, mainly including dormitories and canteens. The beautiful campus environment, comfortable dormitory space, abundant nutritious meals, and reasonable consumer prices enhanced students’ love for the university and let them invest more efforts in learning. It also modified the satisfaction with the major they were studying [47]. In addition, canteen is the biggest life factor that students differ from home to school, the school canteen might have a significant impact on the nutrition, health, growth, and development of students [48]. Good canteen factors provided students with adequate nutrition, provided protection for their physical health, and ensured that students had enough energy to devote themselves to their studies and life, thereby affecting professional satisfaction.

Secondly, employment prospects and understanding of employment status affected professional satisfaction. They were related to personal development prospects and directly affected professional satisfaction [25]. Previous studies showed that the proportion of students dissatisfied with popular majors with good employment prospects was relatively small (about 1–5%), while the proportion of students dissatisfied with unpopular majors was about 30% or more [49]. In China, the scope of enrollment was enlarged, and the number of graduates increased every year. It brought immeasurable employment pressure on college students, and hence the employment situation has become a serious problem. On the whole, the current college graduates’ low starting salary, poor job stability, and increasing proportion of informal employment in their initial employment cannot be ignored [49]. These problems have an impact on students’ professional satisfaction. Therefore, the employment prospects are bright, the employment direction is clearer, the consistency between work and major is higher, and the professional satisfaction of students is higher [6].

The teacher’s teaching level was the third influencing factor for professional satisfaction. Teachers play an indispensable role in the learning process of college students in a transitional period of life [50]. The influencing factors of teacher’s satisfaction with teaching are comprehensively determined by teacher’s teaching attitude, teaching method, teaching level and ability, scientific research level, teacher sex status, teacher image and affinity, and so forth [51]. Research shows that students’ learning satisfaction increases when teachers work hard to prepare lessons and incorporate various diversified teaching elements into lesson plans and teaching practices [52]. Teaching equipment was the key element to perform teaching activities [53]. Advanced teaching equipment was an essential tool for training students who would become medical personnel in the future [54]. Modern technologies such as multimedia, teaching simulation facilities, and virtual simulation facilities provided students with a virtual “hands-on” experience [55, 56], which would stimulate their professional interest and improve their professional satisfaction. Second, the number of study rooms was positively related to student satisfaction [57]. The lack of the study room destroyed the learning atmosphere and reduced the learning enthusiasm of students [58]. It finally decreased the professional satisfaction of students.

The fourth was student major. Similar to other studies, this study also found that public health majors had lower professional satisfaction [59]. In China, the educational system of the public health major is the same as that of clinical medicine. However, its curriculum is a dual-subject learning model, which means that students should complete the medical professional course and the public health professional course within 5 years. Public health students need to complete the basic medical and clinical courses in the first 3 years and then complete the public health professional courses and practice internships in the next 2 years. This implies that the public health professional curriculum is more arduous than the clinical medicine major, and the professional connection incoherent situations ultimately reduce the professional satisfaction of public health students [60–62]. In 1919, Hopkins University established the School of Public Health [63]. However, preventive medicine started late in China. In 1949, China Medical University established the first Department of Public Health [64]. People had a lower level of understanding and did not understand the professional characteristics and work content of public health compared with clinical medicine majors. Public health majors were small majors in medical schools relatively lagged in terms of teacher training, professional curriculum settings, and professional infrastructure construction. Moreover, the salary and job safety in the field of public health were lower than those in the field of clinical medicine [16]. When choosing a major, the proportion of students who were the first choice was relatively low [59], and these inevitably became negative detrimental factors for public health professional satisfaction. In addition, public health professional courses were often started in the 4th grade, whereas the students we surveyed were mainly in the 2.3 grade. The grade distribution of the survey population might have affected the analysis results.

The way of choosing major was the fifth influencing factor for student professional satisfaction. If they chose their own majors independently, their professional satisfaction was relatively higher than that of others [19, 24, 31, 65, 66]. When candidates chose colleges and majors, the suggestions of parents and teachers had a great influence on candidates’ choices [24]. Others often chose majors based on external factors rather than students’ talents or interests. As adults, college students hoped and were able to take responsibility for their future. If their future was decided by others, they were resistant to the major they were studying and even be pessimistic of their major and tired of studying [67]. However, students who chose their majors independently would have a higher degree of recognition of their major, be more interested in learning, and put more efforts into education. These positive factors formed a virtuous circle to increase professional satisfaction [31, 68, 69].

Sixth, different admission results after applying for the National College Entrance Examination also affected students’ professional satisfaction. The study found that the professional satisfaction of students admitted by “first choice” was significantly higher than that of students admitted by “transferred volunteers” [59]. Students’ who admitted by the second choice, third choice, obedience to distribution, and other methods satisfaction decreased in order [10, 19]. On the one hand, this was probably because students had a more thorough understanding of the first choice before applying for the volunteer examination. Non-first-choice students had lower mental preparation and interest in the major they were studying. On the other hand, it was because some majors had not recruited enough people and needed to be adjusted. These majors themselves often had areas that were more difficult to learn or more difficult to find [70]; hence, they could not enhance students’ interest.

The last factor that affected professional satisfaction was sex. Male health professional students had higher professional satisfaction than female ones. First, Tang et al. showed that the sex-specific difference in professional satisfaction might be related to majors. Girls had lower satisfaction with science majors, while boys had lower satisfaction with language and other liberal arts majors [19]. The health profession is a science-based profession, which might be the reason why the professional satisfaction of male health professional undergraduates was higher than that of female ones. Second, the survey found that when choosing a major, male students respected their own hobbies and had a higher degree of understanding of the major they were interested in, while female students were more inclined to refer to the opinions of family members and teachers [38], which indirectly affected the professional of satisfaction of majors of different sexes. In addition, the sex-specific difference in satisfaction might be related to the employment difference between male and female students. Sex-specific discrimination existed in many positions, and girls’ employment expectations were relatively poor, which might also result in girls’ lower professional satisfaction than that of boys [71].

### Strengths and weaknesses

The annual undergraduate enrollment scale was about 252,500 in this survey conducted among undergraduate students of all health professional universities in Hebei province, accounting for 5.75% of the national undergraduate enrollment. The survey scope was wider than that in previous studies. Second, the survey identified the relative factors of professional satisfaction from not only personal characteristics but also school major selection and cognition and university environment aspects. It provided powerful evidence for the construction of universities in the future.

The first limitation of this study was the evaluation of professional satisfaction. In this study, professional satisfaction was a subjective evaluation result. It lacked objective evaluation, leading to bias in the study. Second, the majors with a small number of students were combined with other related majors during random sampling. It might have resulted in the lack of investigation of some majors and hence affected the results.

## Conclusions

The professional satisfaction of health professional undergraduates in Hebei province is high. Adjusting the personal characteristics, the study major and university environment were influencing factors for professional satisfaction. Efforts should be made at the university level to increase the professional satisfaction of students. Universities should strengthen the publicity of majors before enrollment and conduct the employment guidance course after enrollment to increase the understanding of the major. At the same time, universities should improve the campus environment and increase the investment in infrastructure construction to create a more harmonious and comfortable learning and living environment for students.

## Data Availability

The datasets used and/or analyzed in the present study are available from the corresponding author on reasonable request.
